# Progress and Challenges in Integrating Nutritional Care into Oncology Practice: Results from a National Survey on Behalf of the NutriOnc Research Group

**DOI:** 10.3390/nu17010188

**Published:** 2025-01-05

**Authors:** Francesca De Felice, Silvia Malerba, Valerio Nardone, Viola Salvestrini, Natale Calomino, Mario Testini, Virginia Boccardi, Isacco Desideri, Carolina Gentili, Raffaele De Luca, Luigi Marano

**Affiliations:** 1 Radiation Oncology, Policlinico Umberto I, Department of Radiological, Oncological and Pathological Sciences, “Sapienza” University of Rome, 00042 Rome, Italy; francesca.defelice@uniroma1.it (F.D.F.); carolina.gentili@uniroma1.it (C.G.); 2 Department of Precision and Regenerative Medicine and Ionian Area, University of Bari “Aldo Moro”, 70110 Bari, Italy; s.malerba10@studenti.uniba.it (S.M.); mario.testini@uniba.it (M.T.); 3 Department of Precision Medicine, University of Campania “L. Vanvitelli”, 80131 Naples, Italy; valerio.nardone@unicampania.it; 4 Radiation Oncology Unit, Azienda Ospedaliero-Universitaria Careggi, University of Florence, 50110 Florence, Italy; viola.salvestrini@unifi.it (V.S.); isacco.desideri@unifi.it (I.D.); 5 Department of Medicine, Surgery and Neurosciences, University of Siena, 53100 Siena, Italy; natale.calomino@unisi.it; 6 Division of Gerontology and Geriatrics, Department of Medicine and Surgery, University of Perugia, 06123 Perugia, Italy; virginia.boccardi@unipg.it; 7 Department of Surgical Oncology, Istituto di Ricerca e Cura a Carattere Scientifico (IRCCS) Istituto Tumori “Giovanni Paolo II”, 70100 Bari, Italy; raffaele.deluca@oncologico.bari.it; 8 Department of Medicine, Academy of Applied Medical and Social Sciences (AMiSNS), 82-300 Elbląg, Poland; 9 Department of General Surgery and Surgical Oncology, “Saint Wojciech” Hospital, “Nicolaus Copernicus” Health Center, 80-462 Gdańsk, Poland; 10 Department of Surgery, Dnipro State Medical University, 49044 Dnipro, Ukraine

**Keywords:** clinical nutrition, cancer, malnutrition, nutritional assessment, survey, multidisciplinary care

## Abstract

Introduction: Nutritional care is a cornerstone of cancer treatment, with the potential to significantly improve patient outcomes by addressing malnutrition and enhancing recovery. Despite growing awareness, the integration of evidence-based nutritional strategies into oncology remains inconsistent. Following a 2020 survey that highlighted critical gaps, the NutriOnc Research Group launched targeted initiatives to raise awareness and improve practices. This study reassesses progress in integrating nutritional care and identifies persistent challenges. Methods: A 29-question survey was conducted in 2024 among 73 early-career oncologists, surgeons, radiation oncologists, and nutrition specialists across Italy. Respondents provided insights into clinical nutrition program availability, multidisciplinary team composition, screening practices, and educational needs. Results: Encouraging advancements were noted, with 80.8% of respondents reporting the presence of clinical nutrition programs, compared to fewer structured efforts in 2020. However, only 26.0% included nutrition specialists in multidisciplinary teams, and barriers such as financial constraints, limited product availability, and the absence of trained professionals persisted. While 82.2% performed nutritional screening, variability in tools and practices highlighted the need for standardization. Strikingly, 98.6% expressed a strong demand for advanced education on clinical nutrition, emphasizing the need for innovative and accessible training programs. Conclusions: This study reveals a landscape of progress tempered by persistent inequities. To close the gap, oncology must embrace standardized protocols, expand access to trained nutrition specialists, and invest in educational initiatives. The findings offer actionable insights for transforming cancer care through nutrition, underscoring its potential to improve survival, quality of life, and overall treatment success.

## 1. Introduction

Cancer is well documented in the literature to induce systemic inflammation syndrome, resulting in metabolic alterations in protein, carbohydrate, and lipid pathways. This metabolic disruption leads to increased catabolism, weight loss, and alterations in energy and nutrient intake, which are further exacerbated by the side effects of treatment, such as nausea, fatigue, and mucositis [1,2]. Adequate nutritional intake, including energy-dense and protein-rich diets, has been shown to mitigate these effects, improve body weight maintenance, and enhance treatment tolerance and overall survival in cancer patients [3]. Nutrition counseling by healthcare professionals plays a critical role in improving outcomes, particularly for patients with gastrointestinal tumors who often experience mechanical or functional impairments. These individuals derive substantial benefits from “immunomodulating nutrition” [4].

Malnutrition significantly impacts patient outcomes, increasing hospital stay durations by 1.5 to 1.7 times [5]. Furthermore, malnourished patients have a higher risk of mortality within one year post-discharge [6]. Since 2016, the Global Leadership Initiative on Malnutrition (GLIM) has introduced diagnostic criteria for malnutrition, which are applicable to conditions such as cachexia [7]. Clinical nutrition has become an integral component of modern cancer surgery, delivered by a multidisciplinary team that includes oncologists, surgeons, and radiation oncologists [8]. However, despite its importance, nutritional assessment remains underutilized and poorly integrated into surgical oncology departments across Europe [9].

The first multidisciplinary survey by the NutriOnc Research Group [10] highlighted a lack of adherence to nutritional assessment practices, even though they are emphasized in European Society for Clinical Nutrition and Metabolism (ESPEN) guidelines. In response, a targeted program focusing on training and raising awareness about nutritional issues in cancer care has gained traction [11]. To address these gaps, the NutriOnc Research Group has planned various initiatives, including webinars, workshops, and a three-day series of clinical case discussions involving young doctors. These efforts aim to enhance the understanding of significant nutritional challenges in oncology, including the high prevalence of malnutrition and cachexia among cancer patients, which adversely impact treatment outcomes, recovery, and overall survival. Additionally, these initiatives target the structural and systemic barriers that hinder the integration of clinical nutrition into multidisciplinary oncology care, such as limited access to trained nutrition specialists, variability in nutritional assessment practices, and financial and logistical constraints in healthcare institutions.

Building on the findings of a 2020 survey, this study aims to re-evaluate the integration of nutritional care within oncology practices in Italy. It also assesses the impact of various initiatives implemented to raise awareness and improve practices in clinical nutrition.

Specifically, the study evaluated healthcare professionals’ awareness, adoption, and application of evidence-based nutritional interventions, with a focus on the availability of clinical nutrition programs, the implementation of nutritional assessments, multidisciplinary team composition, and the use of screening tools. Furthermore, it aimed to identify persistent barriers to effective nutritional management and explore the educational needs required to address gaps in knowledge and enhance the delivery of nutritional care in oncology.

## 2. Materials and Methods

### 2.1. Survey Design

In 2020, the younger members of the Association of Medical Oncology (AIOM), Italian Association of Radiotherapy and Clinical Oncology (AIRO), Italian Society of Surgical Oncology (SICO), and Italian Society of Artificial Nutrition and Metabolism (SINPE) collaborated to establish shared research objectives in the context of the NutriOnc Research Group. This initiative aimed to develop multidisciplinary strategies to enhance the quality of life for cancer patients. In 2024, the NutriOnc Research Group set up an anonymous electronic questionnaire on SurveyMonkey^©^ (SVMK Inc, One Curiosity Way, San Mateo, CA, USA) to gather insights from medical oncologists, surgeons, radiotherapists, nutrition specialists, and trainees aged 40 years or younger who were members of this organization. It is an update of the previous questionnaire performed in 2022, which primarily focused on identifying baseline practices and gaps in nutritional care [10]. The updated questionnaire is designed to provide a more comprehensive evaluation of progress in clinical nutrition integration and to address specific barriers identified in the earlier survey, such as limited standardization of nutritional assessments and the underrepresentation of nutrition specialists in multidisciplinary teams.

The questionnaire consists of 29 multiple choice questions (Q), divided into four key sections: 1. Knowledge and Practices in Nutritional Management; 2. Nutritional Screening and Assessment Timing; 3. Nutritional Treatment and Post-Hospital Care; and 4. Immunonutrition (IMN) and Educational Needs (Supplementary Material; Table S1). The questions were developed based on the findings of the 2020 survey conducted by the NutriOnc Research Group and were reviewed by a multidisciplinary team of oncologists, surgeons, and nutritionists [10]. To maintain consistency and comparability, several questions were directly adapted from the 2020 survey [10], while new items were added to address emerging challenges and trends.

#### 2.1.1. Section 1: Knowledge and Practices in Nutritional Management

Use of internal protocols and the involvement of nutritionists or dieticians within multidisciplinary teams, including medical nutritionists, oncologists, surgeons, radiotherapists, and trained nurses.

#### 2.1.2. Section 2: Nutritional Screening and Assessment Timing

Timing and oversight of nutritional evaluations, the administration of assessments in both pre- and in-hospital settings, and the types of screening tools employed.

#### 2.1.3. Section 3: Nutritional Treatment and Post-Hospital Care

Management of home therapeutic plans, the types of nutritional therapies recommended, and common challenges faced by patients.

#### 2.1.4. Section 4: Immunonutrition (IMN) and Educational Needs

Participants’ knowledge of immunoenhanced nutrition and suggestions for further educational initiatives.

### 2.2. Survey Dissemination

Young professionals were encouraged to participate by completing modules relevant to their clinical practices. The questionnaire was hosted in a secure section of the NutriOnc Research Group website, accessible through a direct email link sent individually to eligible participants via SurveyMonkey^®^ (SVMK Inc, One Curiosity Way, San Mateo, CA, USA). The survey remained open from 14 May 2024 to 30 July 2024, and all responses were submitted anonymously.

### 2.3. Statistical Analysis

The collected responses were analyzed descriptively, without a pre-calculated sample size for hypothesis testing. Statistical summaries of both continuous and categorical data were used to describe respondents’ characteristics and their answers to specific items. Results were compared among the three professional societies. As the analysis was exploratory in nature, no adjustments were made for multiple comparisons.

## 3. Results

### 3.1. Description of the Cohort

**Figure 1:**
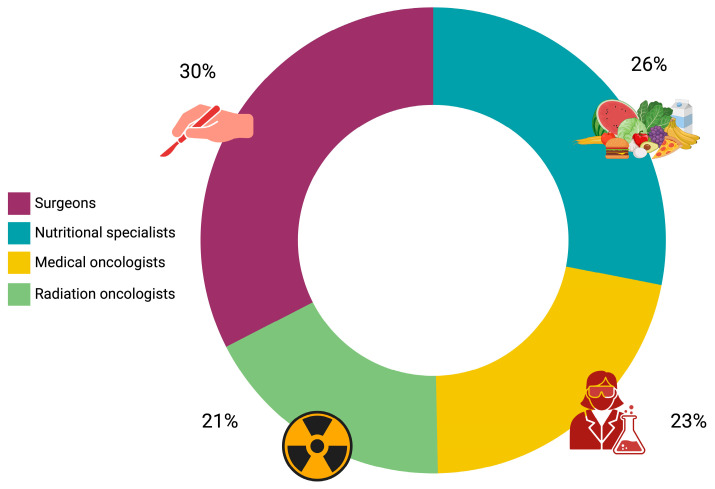
Pie chart visualization of professional distribution of survey respondents by specialty.

A total of 350 healthcare professionals from the target population were invited to participate in the survey, of which 73 (20.8%) responded. Among the 73 respondents, 22 (30.1%) were surgeons, 19 (26.0%) were nutrition specialists, 17 (23.3%) were medical oncologists, and 15 (20.6%) were radiation oncologists (Figure 1).

**Figure 2:**
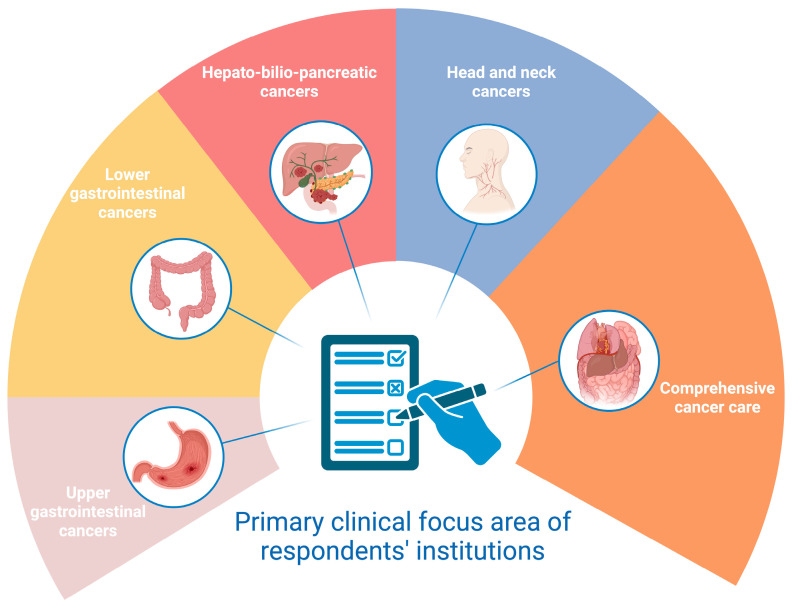
Prevalence of primary clinical focus areas among respondents’ institutions.

The participating physicians represented various regions across Italy, with the majority coming from Emilia-Romagna (*n* = 16; 21.9%) and Veneto (*n* = 10; 13.7%). Most respondents (*n* = 35; 48.0%) were early-career specialists with less than five years of post-residency experience and were employed in high-volume centers. Additionally, 33 (45.2%) physicians reported that their daily clinical activities primarily focused on managing upper and lower gastrointestinal cancers, while 8 (11.0%) specialized in head and neck cancer care (Figure 2).

### 3.2. Knowledge Regarding Nutritional Management Modalities

Fifty-nine (80.8%) physicians reported that a clinical nutrition program was established in their institution. Conversely, 14 (19.2%) stated that they adopted nutritional recommendations based on their personal judgment. This was primarily attributed to the absence of a clinical nutrition specialist (12.3%), poor communication (11.0%), or lack of financial support (5.5%). Nearly all physicians indicated adherence to the Enhanced Recovery After Surgery (ERAS) guidelines.

### 3.3. Nutritional Status Evaluation

Overall, patients with a cancer diagnosis are discussed with a multidisciplinary board in most cases (*n* = 71; 97.3%), with a nutrition specialist included as a member in 19 instances (26.0%). Fifty-two physicians (71.2%) reported that nutritional screening is conducted during routine visits, typically by medical or radiation oncologists (28.8%), nutrition specialists (26.0%), surgeons (21.9%), physicians with clinical expertise in nutritional assessment (13.7%), or case-manager nurses (9.6%).

Sixty respondents (82.2%) indicated familiarity with various tools for conducting nutritional screenings, including the Malnutrition Universal Screening Tool (MUST) (68.5%), Nutritional Risk Screening 2002 (NRS-2002) (38.4%), Malnutrition Screening Tool (MST) (16.4%), Mini Nutritional Assessment Short-Form (MNA-SF) (32.9%), Global Leadership Initiative on Malnutrition (GLIM) criteria (26.0%), Bioelectrical Impedance Vector Analysis (BIVA) (30.1%), and computed tomography (CT) scans of the L3 vertebral body (15.1%).

When an abnormal nutritional screening result is identified, most respondents (67, 91.8%) recommended further nutritional evaluation. This evaluation primarily relied on hematochemical indices, such as serum albumin, prealbumin, creatinine, iron levels, 3-methylhistidine, cholesterol, and total lymphocyte count (57.5%), food diaries (56.2%), and the MNA-SF (39.7%). Nearly all physicians (*n* = 65; 89.0%) generally agreed on the importance of periodic nutritional status assessments at diagnosis and prior to both surgery and chemoradiotherapy.

### 3.4. Treatment Issues

When nutritional support is required, 53 physicians (72.6%) reported that nutritional prescriptions are provided by nutrition specialists. The remaining cases involved medical oncologists (20.5%), surgeons (5.5%), and radiation oncologists (1.4%).

The primary challenges in nutritional management were identified. Clinically, nausea and vomiting (76.7%), fatigue (34.3%), dysphagia (32.9%), malabsorption (31.5%), and mucositis (22.0%) were the main factors negatively impacting optimal nutritional care. Additionally, reimbursement issues and the availability of nutritional products in hospital pharmacies were cited as significant barriers to treatment.

For managing malnutrition (defined as weight loss > 5% within the last three months), physicians favored oral nutritional supplements (38.4%) and nutritional counseling (32.9%) over enteral nutrition (2.8%) and parenteral nutrition (2.8%).

A total of 34 physicians (46.6%) indicated that supplementary nutrition should be recommended for patients undergoing radiotherapy with or without concomitant chemotherapy. The most commonly suggested supplements included arginine (45.2%), omega-3 fatty acids (41.1%), vitamins/antioxidants (41.1%), glutamine (32.9%), whey protein (20.6%), and RNA (21.9%).

Sixty-four physicians (87.7%) reported being familiar with immunonutrition, which is routinely prescribed perioperatively (61.6%), before surgery (46.6%), and during chemoradiotherapy (21.9%).

### 3.5. Evaluation of New Strategies to Improve Nutritional Knowledge

In total, 72 out of 73 (98.6%) physicians, independent of their knowledge regarding immunonutrition, would be interested in participating in educational and training programs to increase awareness of clinical nutrition topics. Suggested strategic priorities included thematic webinars (74.0%) and evidence-based literature (41.1%).

## 4. Discussion

The current survey highlights significant advancements and persistent gaps in the awareness and implementation of nutritional care among oncology professionals in Italy, compared to the previous survey conducted in 2020 [10]. This analysis provides a comprehensive perspective on how multidisciplinary efforts, increased educational initiatives, and evolving clinical practices over the past three years have influenced knowledge and attitudes toward clinical nutrition in oncology.

One notable improvement is the broader inclusion of nutrition specialists and the increased prevalence of defined clinical nutrition programs. Despite these advancements, many patients continue to experience inadequate energy and nutrient intake during treatment, which contributes to weight loss, poor treatment tolerance, and increased complications. Studies have demonstrated that maintaining energy balance and addressing specific nutritional deficiencies can significantly improve treatment outcomes, reduce hospital stays, and enhance the quality of life in oncology patients [2,3,4,5]. Nutritional interventions, such as personalized diet plans incorporating protein-rich foods and supplements, should be prioritized to address these challenges [9,10,11,12]. In this survey, 80.8% of respondents reported the presence of a clinical nutrition program within their institutions, reflecting a positive shift from the less structured approaches observed previously. This trend aligns with the emphasis on adhering to ERAS guidelines, which continue to serve as a cornerstone of nutritional management in cancer care. However, while awareness of ERAS has improved, its consistent implementation remains uneven, particularly in institutions lacking nutritionists or facing financial constraints. These disparities underscore the need for stronger institutional support and resource allocation to ensure the uniform application of evidence-based nutritional protocols.

The high utilization of multidisciplinary boards in patient care, reported by 97.3% of respondents, marks another positive trend. This improvement highlights the growing integration of collaborative decision-making processes in oncology. Nevertheless, the inclusion of nutrition specialists in these boards remains limited, with only 26.0% of respondents indicating their presence. This low representation is concerning, as evidence suggests that the involvement of trained nutrition specialists in multidisciplinary teams is associated with improved patient outcomes, including better nutritional status, reduced treatment-related complications, and enhanced quality of life [13,14]. These results underscore the critical need to prioritize the integration of nutritional expertise into oncology care, as highlighted later in this discussion. These findings echo previous challenges, where the absence of trained nutritionists was a major obstacle. Although structural frameworks for collaboration exist, the underrepresentation of nutritional expertise in these settings reveals an ongoing gap that must be addressed to ensure comprehensive patient care.

Recent literature continues to emphasize the critical role of nutritional care in oncology. A growing body of evidence demonstrates a clear correlation between nutritional status and clinical outcomes, including all-cause mortality in cancer survivors [12]. This reinforces the importance of comprehensive nutritional assessments in enhancing survival rates and quality of life. Moreover, technological advancements, such as artificial intelligence (AI), are being explored to monitor and follow up on the nutritional status of oncological patients [15]. These innovations offer promising solutions to bridge gaps in care and standardize follow-up processes.

Further contributions to the field come from the American Society for Parenteral and Enteral Nutrition (ASPEN), which conducted a systematic review and recommended validated malnutrition screening tests for cancer patients [16]. These recommendations align with findings from the current survey, which highlight increased awareness and use of screening tools such as the MUST, NRS-2002, and MNA-SF. Despite this progress, significant variability persists regarding the professionals responsible for conducting these assessments. Often, surgeons and medical oncologists assume this role instead of dedicated nutritionists, which can have significant implications for the quality of assessment and patient outcomes. While these professionals are skilled in their primary disciplines, they may lack the specialized training required to perform comprehensive nutritional evaluations, potentially leading to underdiagnosis or suboptimal management of malnutrition. This gap in expertise could result in delayed interventions, poorer treatment tolerance, and increased complications, emphasizing the need for integrating trained nutritionists into oncology teams.

Several recent studies have addressed the unmet needs of cancer patients regarding malnutrition assessment and its impact on treatment outcomes across various contexts [17,18,19,20,21]. These studies underscore the necessity of prioritizing malnutrition management to improve treatment tolerance, reduce complications, and enhance overall survival. Financial toxicity has emerged as a critical barrier to accessing nutritional counseling and advanced therapies for cancer patients. Potential solutions include incorporating nutritional counseling into national cancer treatment guidelines, advocating for insurance coverage of advanced therapies, and incentivizing healthcare institutions to include trained nutritionists in multidisciplinary teams. These systemic changes, supported by policy-level interventions and financial mechanisms, could reduce disparities in care and ensure that all patients receive comprehensive and timely nutritional support [22].

Comparative data from other surveys offer valuable benchmarks. For instance, the European Society of Surgical Oncology—Young Surgeons and Alumni Club (ESSO-EYSAC) analysis reported a malnutrition screening rate of 38% among surgical oncologists [23], while the Associazione Italiana di Ematologia ed Oncologia Pediatrica (AIEOP) analysis highlighted the need for unified recommendations to improve nutritional care in pediatric oncology [24]. These findings resonate with the gaps identified in the current survey and emphasize the need for consistent protocols and stronger interdisciplinary collaboration across specialties.

In this context, the ongoing efforts of Italian professional communities, such as the NutriOnc Research Group, are pivotal. Over the past few years, this group has organized several congresses and events to raise awareness of malnutrition among oncology professionals. These initiatives have significantly contributed to the progress observed in the current survey and should be expanded further. The integration of more educational formats, such as webinars and interactive workshops, could enhance the reach and impact of these efforts. Engaging governance and institutional stakeholders is also crucial to ensuring that malnutrition management becomes a prioritized component of oncology care. Policies and funding mechanisms must address financial toxicity to reduce disparities and improve access to high-quality nutritional care.

Despite these positive developments, challenges in nutritional management persist. Common barriers, including nausea, vomiting, dysphagia, and limited availability of nutritional products, remain consistent with the findings of the previous survey.

Furthermore, the preference for oral nutritional supplements and counseling over enteral or parenteral nutrition reflects both a tendency toward less invasive interventions and a lack of resources or training in advanced nutritional therapies. To address the barriers identified in this survey, several strategies can be implemented to improve the integration of nutritional care into oncology practice. First, institutional prioritization of nutritional care is essential, requiring the incorporation of clinical nutrition into standard oncology care pathways and securing dedicated funding to support these programs [25,26]. Simultaneously, the training and education of healthcare professionals should be enhanced by developing comprehensive modules that improve the knowledge and practical application of clinical nutrition, potentially embedding these into continuing medical education (CME) requirements. Standardized guidelines tailored to the oncology setting also play a crucial role. Collaborative efforts with organizations such as ESPEN and ERAS can help establish and disseminate protocols that ensure consistency in nutritional management across diverse clinical settings. In addition, including trained nutrition specialists as part of multidisciplinary teams can enhance the personalization of nutritional interventions, optimizing patient outcomes and improving overall care quality. Policy-level changes are equally important. Advocacy for national policies and insurance coverage to mitigate financial barriers could increase access to advanced nutritional therapies and supplements, addressing one of the most significant obstacles identified in this survey. Finally, empowering patients through accessible educational resources, such as workshops and online platforms, can enhance their understanding of the importance of nutritional care and encourage better adherence to dietary recommendations tailored to their specific needs [27].

The current survey faces certain limitations compared to the previous one, which may affect the interpretation of its findings. The smaller response cohort—73 physicians compared to 215 in the earlier survey—reduces the generalizability of the results, as the reported practices may not fully represent the broader oncology community in Italy. Furthermore, the geographic distribution of respondents was more concentrated, with a focus on regions such as Emilia Romagna and Veneto. This regional concentration may disproportionately emphasize practices common in high-volume centers, underrepresenting challenges faced by smaller or less-resourced institutions. Consequently, findings related to resource availability and clinical nutrition implementation may primarily reflect the experiences of well-equipped facilities, limiting their applicability to all settings. Despite these constraints, the insights gained from this survey provide valuable direction for addressing gaps in nutritional care.

Additionally, the reduced number of responses constrained the feasibility of conducting detailed subgroup analyses across disciplines. This limitation affects the ability to identify profession-specific gaps and develop tailored recommendations for surgeons, medical oncologists, and other professionals involved in nutritional care. Future studies should prioritize a larger, more diverse sample to facilitate robust subgroup analyses, enabling targeted interventions that address the unique challenges faced by each discipline in integrating nutritional care into oncology practice.

Moreover, the reliance on self-reported data introduces the potential for response bias, which may impact the accuracy of reported practices and perceptions. This bias could lead to an overestimation of positive practices or an underrepresentation of challenges.

Despite these limitations, this study provides valuable insights into key barriers to integrating nutritional care into oncology practice and highlights critical gaps that must be addressed. It serves as a foundation for future, larger-scale research to validate these findings, enhance geographic and professional representation, and develop actionable strategies to advance nutritional care integration across diverse settings.

While the survey touches upon differences between surgeons, oncologists, and radiation oncologists, the limited data restrict the ability to perform a deeper comparative breakdown, which is critical for identifying discipline-specific gaps. Future surveys should aim to address these shortcomings by engaging a larger and more diverse participant pool and exploring granular data across professional roles and institutional contexts.

The findings of this survey, juxtaposed with recent advancements and ongoing challenges, underscore the critical need for targeted interventions to address malnutrition in cancer care. Personalized nutrition is particularly critical, as the nutritional requirements of patients vary based on the type and stage of cancer, treatment modality, and individual metabolic and physiological conditions [2,3,4]. For example, patients with gastrointestinal cancers often face malabsorption issues and may require higher protein and caloric intake to maintain energy balance, while those undergoing chemoradiotherapy may benefit from specialized diets rich in antioxidants and immune-modulating nutrients to counteract oxidative stress and inflammation [21,22,23,24,25,26,27,28,29]. Tailored dietary plans, developed in collaboration with trained nutritionists, can address these specific needs, improve treatment tolerance, reduce complications, and enhance quality of life [25,26,27,28,29].

These include standardizing nutritional protocols, increasing access to nutritionists, leveraging AI-driven solutions for nutritional follow-up [30], and addressing financial and institutional barriers. Expanding initiatives such as congresses, events, and webinars to enhance awareness and knowledge of malnutrition is vital. Efforts should also focus on engaging governance and institutions to prioritize malnutrition as a key component of cancer care, ensuring policies and funding mechanisms that reduce financial toxicity. By addressing these gaps and aligning strategies with the ongoing work of multidisciplinary groups like NutriOnc, the field of oncology nutrition can achieve more comprehensive and equitable patient care.

## 5. Conclusions

Our findings underscore substantial progress in the integration of nutritional care within oncology practice in Italy, evidenced by the increased presence of clinical nutrition programs and the inclusion of nutrition specialists in multidisciplinary teams. However, inconsistencies in the implementation of nutritional assessments and the underrepresentation of nutrition specialists remain significant challenges, particularly in resource-limited settings. Financial barriers and the limited availability of nutritional products further hinder optimal care. This survey highlights an urgent need for targeted educational initiatives to address gaps in knowledge and enhance the capacity of oncology professionals to meet the nutritional needs of cancer patients effectively. As a projection of this study, we propose the development of a national or international consensus on nutritional care in oncology. Such a consensus would provide standardized, evidence-based guidelines tailored to different cancer types and patient profiles, addressing barriers such as resource variability and lack of trained specialists. By fostering collaboration among multidisciplinary teams and incorporating insights from experts in oncology and nutrition, this initiative could significantly enhance the quality of nutritional care and improve patient outcomes. By improving the standardization of nutritional protocols, expanding access to trained nutritionists, and advocating for institutional and policy-level support, patient care can be significantly enhanced. Such measures have the potential to improve survival, reduce treatment-related complications, and enhance quality of life for oncology patients, particularly those at higher risk of malnutrition.

## Data Availability

Data are contained within the article and supplementary materials.

## Supplementary Materials

The following supporting information can be downloaded at https://www.mdpi.com/article/10.3390/nu17010188/s1: Table S1: Sections and items of the questionnaire.
