# Supplementary material for: Progress and Challenges in Integrating Nutritional Care into Oncology Practice: Results from a National Survey on Behalf of the NutriOnc Research Group
Source: Nutrients. 2025 Jan 5;17(1):188. doi: 10.3390/nu17010188 (PMC11722632; doi:10.3390/nu17010188)
Supplement: Supplementary file 1 [file nutrients-17-00188-s001.zip › nutrients-3399978-supplementary.pdf]

Table S1. Sections and items of the questionnaire

Participants’ demographics and institutional volumes				
Q1. Respondent’s general information	-	-	-	-
Q2. Society Affiliation	SICO	AIOM	AIRO	SINPE
Q3. Geographic distribution	North	Center	South	
Q4. Field of specialization	Medical oncologist	Surgical oncologist	Radiation oncologist	Nutritionist
Q5. Role of partecipants	Resident	Consultant (0-5 Y)	Consultant (5-10 Y)	
Q6. Primary Clinical Focus of Respondents' Institutions	Gastroesophageal cancer	Colorectal cancer	Head-neck cancer	Epato-biliary-pancreatic cancer
Q7. How many patients with localized or locally advanced gut disease are treated annually at your center?	Gastroesophageal cancer<br><20<br>>20 <40<br>>40	Colorectal cancer<br><60<br>>60 <100<br>>100	Epato-biliary-pancreatic cancer<br><20<br>>20 <40<br>>40	
Q8. How many patients with localized or locally advanced head-neck disease are treated annually at your center?	Head-neck cancer<br><20<br>>20 <40<br>>40			

SECTION 1: Knowledge and Practices in Nutritional Management					
Q9. Is there a specific internal protocol for clinical nutrition of patients at the center where you work?	Yes (Eras)	Yes (Other)	No		
Q10. If not, do you know why?	Lack of nutritionists	No interaction with the nutrition group	Lack of financial support	Lack of training events	
Q11. Are there nutritional protocols for the malnourished cancer patient who undergoes neoadjuvant therapy?	Yes	No	Only in case of Gastro-Esophageal tumors	Only in case of Hepato-bilio-pancreatic tumor	Only in case of Colorectal tumors
Q12. Is there a multidisciplinary team in your centre that evaluates cancer patients?	Yes	No			

Table S1. Sections and items of the questionnaire

Q13. If there is no nutritionist: do you have a consultant?	Yes	No

SECTION 2: Nutritional Screening and Assessment Timing

Q14. Does your center carry out nutritional screening of patients?	Yes	No					
Q15. Who is responsible for nutritional screening of patients if a nutritionist/dietitian is not available?	Surgical team	Nutrition specialist					
Q16. f yes, when is it performed?*	First visit/ at the time of diagnosis	Before the surgical treatment	After the surgical treatment	Before the radio-chemotherapy treatment	After the radio-chemotherapy treatment	Always during therapeutic period of care	
Q17. Who is responsible for the nutritional screening of patients if there is no nutritionist/dietician?	A physician with experience in nutritional assessment	Nurse Case Manager	The surgical team	Medical Oncologist/Radiation oncologist			
Q18. Do you know any nutritional screening method?*	Yes	No					
Q19. f yes, which of this?	Yes (Malnutrition Universal Screening Tool, MUST)	Yes (Malnutrition Screening Tool, MST)	Yes (Nutritional Risk Screening 2002, NRS-2002)	Yes (Mini Nutritional Assessment Short-Form, MNA-SF)	Yes (GLIM criteria)	Yes (instruments: Bioelectrical impedance vector analysis, BIVA)	Yes (instruments: CT at L3 level)
Q20. If yes, when is it perfomed?*	First visit/ at the time of diagnosis	Before the surgical treatment	After the surgical treatment	Before the radio-chemotherapy treatment	After the radio-chemotherapy treatment	Always during therapeutic period of care	
Q21. If a nutritional screening reveals an abnormal result, is a nutritional assessment then performed?	Mini Nutritional Assessment Short-Form, MNA-SF	Subject Global Assessments, SGA	Score patient-generated Subject Global Assessment, PG-SGA	GLIM criteria	Instruments: Bioelectrical impedance vector analysis, BIVA	Instruments: CT at L3 level	Instruments: Blood tests/PCR

Table S1. Sections and items of the questionnaire

*More than one answer was eligible.

SECTION 3: Nutritional Treatment and Post-Hospital Care										
Q22. If nutritional support is required to be administered at the patient's home, who is responsible for activating the therapeutic plan?	Medical oncologist	Surgical oncologist	Radiation oncologist	Nutritionist						
Q23. In the case of a malnourished patient (e.g., weight loss >5% within less than 3 months), what type of nutritional therapy is typically provided?	Nutritional Counseling	Integration per os	Enteral via PEG	Associated/ mixed (os/parenteral)	Partial/ total parenteral	Other				
Q24. What challenges do you encounter during the delivery of nutritional therapy?*	Supply and/or Refundability of the product	Absence of a therapeutic plan in the regional Risk Assessment Document	Absence of a nutritionist in the ward	Mucositis	Asthenia/ loss of appetite	Dysphagia	Nausea	Vomiting	Malabsorption	Other

*More than one answer was eligible.

Table S1. Sections and items of the questionnaire

SECTION 4: Immunonutrition (IMN) and Educational Needs							
Q25. Do you think there are essential substrates for the nutritional support of patients treated with radiotherapy?	Yes	No					
Q26. Are you familiar with immunonutrition?	Yes	No					
Q27. If YES, can you indicate when it is prescribed? *	Perioperative	Preoperative	In Radio/Chemotherapy				
Q28. Are there any topics related to nutrition in oncology that you would like to investigate? *	Clinical impact of immunonutrition	Results of immunonutrition in patients undergoing neoadjuvant treatment	Surgical outcomes in immunotreated patients	Cost-effectiveness analysis of immunonutrition			
Q29. How do you wish to receive information (indicate the methods you prefer)? *	Specific webinar	Clinical work presentation	Specific webinar	Clinical work presentation	Podcast	Infographic card	Slide set
